# Reviewed article: Silva JRS, Arruda RG, Silva BO, Menezes ESB, Diniz CMM, Gomes JPA, Araújo BC, Rêgo MJBM, Pitta MGR, Pereira MC. High prevalence of respiratory diseases: a population-based ecological study, Sertão do Araripe, 2008-2019. Epidemiol Serv Saude. 2025:34;e20240519

**DOI:** 10.1590/S2237-96222025v34e20240519.b

**Published:** 2025-09-15

**Authors:** Aluísio Oliveira

**Affiliations:** 1 Instituto Federal de Educação, Ciência e Tecnologia do Sudeste de Minas Gerais, Juiz de Fora, MG, Brazi

## Peer review B

## Questionnaire

Does the manuscript contain new and significant information to justify publication? Yes

Does the Abstract (Summary) clearly and accurately describe the content of the article? Yes

Is the problem significant and concisely stated? Yes

Are the methods described comprehensively? No

Are the interpretations and conclusions justified by the results? No

Is adequate reference made to other work in the field? No 

## Manuscript Structure

Length of article is: Too short

Number of tables is: Adequate

Number of figures is: Adequate

Please state any conflict(s) of interest that you have in relation to the review of this paper. None

Interest: Good

Quality: Good

Originality: Good

Overall: Good

Recommendation: Major Revision

## Comments

O trabalho apresentado é de grande relevância e aborda um tema pertinente para os estudos da área de saúde no pais. Contudo, seria essencial incluir um maior detalhamento da população amostrada para aumentar a precisão das análises e das conclusões. Por exemplo, é necessário esclarecer a distinção entre os grupos diretamente expostos, como os trabalhadores que lidam com poeira de gesso e fumaça resultante da queima de biomassa (lenha) utilizada na calcinação, e os habitantes das cidades que podem estar indiretamente expostos. Essa diferenciação contribuiria para um entendimento mais aprofundado dos impactos em cada segmento populacional e fortaleceria a validade científica do estudo. Ademais, cabe destacar a necessidade de uma revisão linguística do texto.

